# Editorial: Microbial Chain Elongation- Close the Carbon Loop by Connecting-Communities

**DOI:** 10.3389/fbioe.2022.894490

**Published:** 2022-07-08

**Authors:** David P. B. T. B. Strik, Ramon Ganigué, Largus T. Angenent

**Affiliations:** ^1^ Environmental Technology, Wageningen University and Research, Wageningen, Netherlands; ^2^ Center for Microbial Ecology and Technology, Ghent University, Ghent, Belgium; ^3^ Center for Advanced Process Technology for Urban Resource Recovery (CAPTURE), Gent, Belgium; ^4^ Environmental Biotechnology Group, Center of Applied Geosciences, University of Tübingen, Tübingen, Germany

**Keywords:** chain elongation, product inhibition, branched carboxylates and alcohols, oxygen contamination, dairy manure, thin stillage, downstream processing, pertraction

## 1 Introduction

### 1.1 Chain Elongation: Discoveries That Evolve in Various Biorefinery Platforms

Biomass, waste (waters), and captured CO_2_ are crucial inputs to close the carbon-loop of our society. To enable the “Circular Economy” these resources should be converted into chemicals and circular products (European Commission, 2015). Micro-organisms and their enzymes are especially suited for this as they are robust, versatile, and cheap catalysts. They can convert a wide variety of resources from simple C1 compounds, such as CO, CO_2,_ or formic acid, as well as complex materials such as cellulose (Arslan et al., 2016). It is well known that micro-organisms can convert complex materials via anaerobic digestion into biogas, primary consisting out of CH4 and CO_2_ (Lettinga, 1995). Once methanogenesis is inhibited, the anaerobic digestion process can be turned into a so called “mixed-acid fermentation” or “methane-arrested anaerobic digestion” leading to accumulation of mainly short-chain carboxylic acids such as acetate (and smaller amounts of lengthier carboxylic acids). This fermentation process was coupled to further downstream processing to produce mixed alcohols via the so called MixAlco process (Holtzapple et al., 1997; Holtzapple and Granda, 2009).

Several microbial pathways, such as the reversed beta-oxidation, have been shown to allow carbon-chain elongation (Seedorf et al., 2008). This way, short chain carboxylic acids, from, for example, the mixed acid fermentation, could be further elongated to mainly medium chain carboxylic acids such as caproic acid and even caprylic acid. In 1995, Kenealy et al. used a co-culture to convert cellulose and ethanol to caproic acid (Kenealy et al., 1995); Steinbusch et al. used an open culture to elongate ethanol and acetic acid up to caprylic acid (Steinbusch et al., 2011); and Agler et al. also used an open culture to convert ethanol beer into caproic acid with in-line extraction (Agler et al., 2012a). These latter studies demonstrated that by integrating open cultures in bioreactors, one can stimulate chain-elongation processes to develop biotechnological processes, which produce multi-carbon biochemicals such as medium-chain carboxylic acids (Angenent et al., 2016).

**FIGURE 1:**
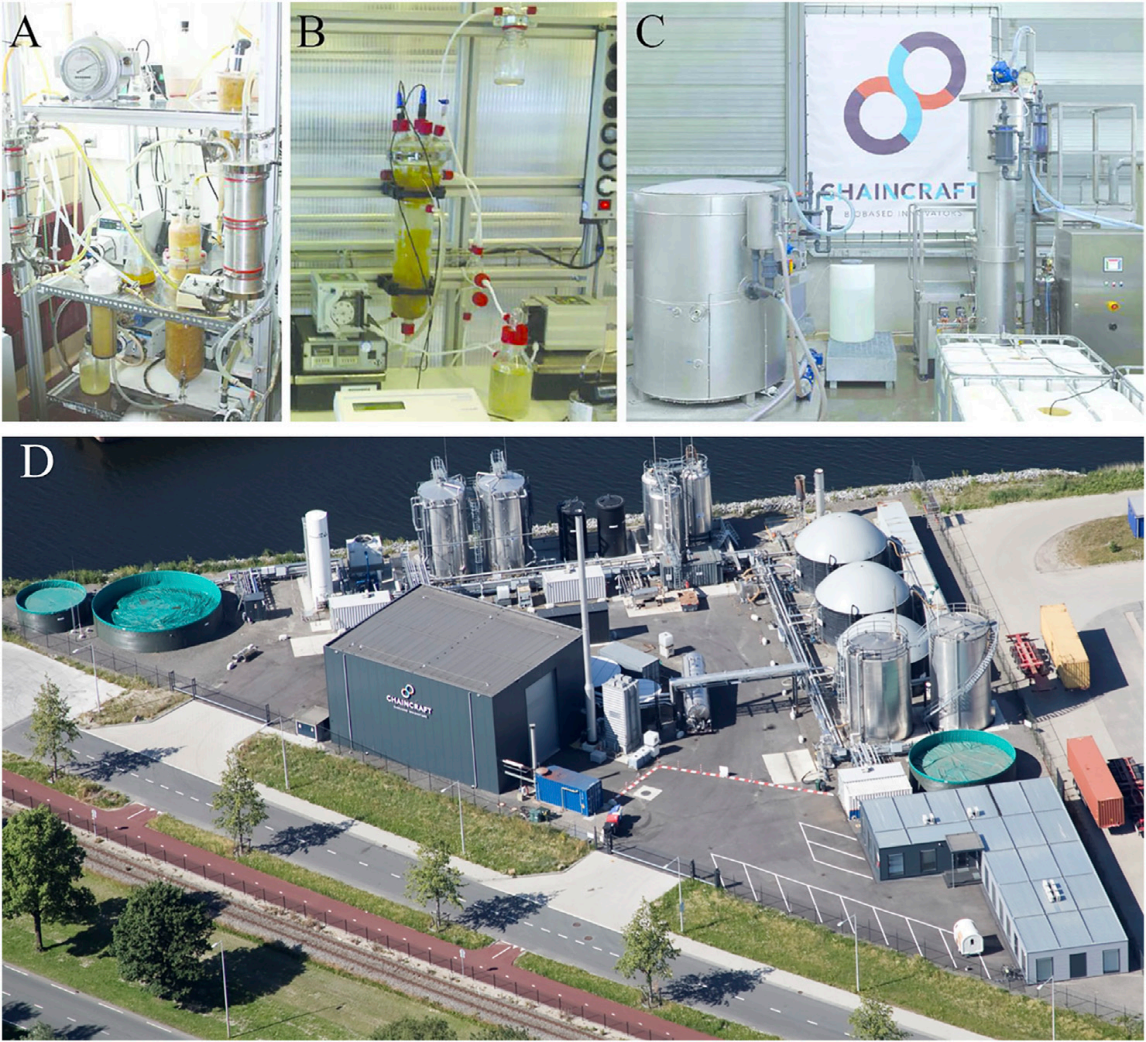
Chain elongation bioreactor developments. Technology Readiness Level (TRL) emerged from lab TRL 2 to 4 **(A,B)** to pilot TRL 5 to 6 **(C)**, and to demonstration TRL 7 to 8 **(D)**: plant operated by ChainCraft, Amsterdam, Netherlands). *ABC: Reprinted with permission from* (Angenent et al., 2016) *with copyright 2016 American Chemical Society. D: kindly provided by ChainCraft.*

Nowadays, multiple bioprocess concepts using a variety of feedstocks are under development, which can lead to the production of valuable multi-carbon biochemicals for (potential) usage in various sectors, including food, feed, agriculture, bioremediation, bioplastic, and biofuel productions. Chain elongation is emerging as a bioprocess connected to various biorefineries (Angenent et al., 2016; Wu et al., 2019a, 2019b). Over the past years, the technology developed from the lab to pilot and reaching a demonstrating technology readiness level (see Figure 1). Currently, ChainCraft is producing liquid and powder based mixtures of dissociated carboxylates for application as animal nutrition and health solution (Chaincraft B.V., 2022). This company is one of several companies world-wide successfully commercializing “Chain Elongation”. The other initiatives include Capro-X, Inc. (Capro-X, 2022), Afyren (2022), Evonik & Siemens (Evonik.com, 2022)., the Capra project in Belgium (Capra project, 2022), and Jager Biotech GmbH (Jager Biotech, 2022) In exigence with this, the first International Chain Elongation Conference (ICEC 2020) was organized bringing 208 participants together on this emerging topic in 2020. Many recorded presentations are online and the book of abstracts is available online in open-access (Strik et al., 2020). Connected to this event, the goal of this frontiers research topic was to bring together a collection of articles on “Microbial Chain Elongation” to provide an inspiring overview of recent advances and future perspectives.

### 1.2 Recent Advances

#### 1.2.1 Bioprocess Development Towards New Products

For chain elongation to become an impactful bioproduction platform, both feedstock utilization and expansion to new products is needed. The ability of wild-type as well as genetically modified organisms in defined or open bioreactor “microbiomes” can be explored. Branched-carboxylic acids represent a new group of chemicals out of these bioreactors. Recently it was shown that the branched MCC iso-caproic acid (i-C6) was formed in considerable amounts (1.4 gL^−1^) when iso-butyric acid (i-C4) and ethanol were used (de Leeuw et al., 2020). This research suggests that formation of other branched MCCs, such isoheptanoic acid (i-C7), could be possible. In this special topic, De Leeuw et al. explored the use of various branched carboxylic acids within an ethanol-based chain elongation process and established that small amounts of 5-methyl-hexanoic acid, likely from 3-methyl-butanoic acid, were produced. The racemic mixture of L/D 2-methyl-butanoic acid did not lead to an elongated product. When isobutyric and isovaleric acid were added simultaneously as substrates, there was a preference for elongation of isobutyric compared to isovaleric acid. Moreover, the corresponding alcohols of both the straight as well as produced branched carboxylic acids were produced. To enable industrial production of branched iso-caproic acid, iso-butyric acid should be available. Fortunately, isobutyric acid formation was observed when methanol-based chain elongation with food-waste was explored (Chen et al., 2017). The promising selectivity to iso-butyric acid was achieved after finding out that pH was key to steering the methanol conversion and microbiome composition (De Leeuw et al., 2020). Moreover, an isolated species from a lactic acid-based chain elongation process was able to produce iso-butyric acid (Liu et al., 2020). In addition, an open culture showed iso-butyric acid formation once specific electric-conductive materials were added within a lactic acid based chain elongation process. Contreras-Dávila et al. (2022) also added nano Zero Valent Iron (nZVI) in a similar bioprocess as described in this topic https://doi.org/10.3389/fbioe.2021.666582. Various effects on the product formation with nZVI as well as by using either D- and/or l-lactic acid substrates were discovered. nZVI enhanced chemical hydrolysis of lactate oligomers present in highly concentrated lactic acid solutions; something which could be exploited to hydrolyze poly lactic acid (PLA) plastics. nZVI created, in a dose-dependent manner, favorable conditions for either chain-elongating or propionic acid producing microbiomes. Several mechanisms could play a role in this, such as, for example, the lowering of the oxidation-reduction potential or provision of extra reducing equivalents. Evidently, metals and other conductive materials can influence microbial communities as demonstrated in anaerobic digestion for biogas production (Liu et al., 2012; Lovley, 2017). Possibly, direct interspecies electron transfer (DIET) could be stimulated by such materials during chain elongation, which is often theorized (Liu et al., 2017; Contreras-Dávila et al., 2022). However, the proof for this mechanism with a chain elongator and the actual stimulation of this in a bioreactor is still elusive. Evidently, the use of metals makes things complex but generally relevant since nZVI application in ethanol-based chain elongation studies also showed several similar effects (Fu et al., 2021).

Contreras-Dávila et al. also evaluated the use of D- and/or l-lactic acid. Earlier work suggested that the chain elongator *M. elsdenii* could produce even-chain carboxylates from d-lactic acid and make odd-chain carboxylates from l-lactic acid (Hino and Kuroda, 1993). From this is was hypothesized that product formation under open-culture conditions would be similarly affected. However, D- and l-lactic acid were both converted to butyric acid while l-lactic acid needed a lag time before chain elongation occurred. It was found that during fermentation both L and d-lactate’s were racemized to a racemic equilibrium (i.e., l-lactic acid was isomerized to d-lactic acid and vice versa). In other open culture fermenters, a low pH (∼<6–6.5) and low lactic acid concentration were shown to be required to prevent propionate formation (Zhu et al., 2015; Kucek et al., 2016; Candry et al., 2020b). In Contreras-Dávila et al. experiments, the pH increased from 5.5. to 7.7 and consequently all lactic acid was consumed. These changing conditions were apparently also not suitable enough to stimulate lactic acid to propionic and acetic acid conversion during the relative short batch period. In another study, Contreras-Dávila et al. found out that supply of propionic and lactic acid at low pH ∼5.5 does enhance chain elongation to heptanoic acid (Contreras-Dávila et al., 2021). Therefore, a strategy to produce odd-MCCA via open cultures could be a train of three separated bioprocesses whereby bioproduced lactic acid is consequently converted into propionic acid and hereafter chain-elongated to MCCAs.

#### 1.2.2 Bioreactor Studies on Kinetics, Inhibition Effects, and Microbial Key-Players

Articles in this special topic also used bioreactors as a tool to study how operating conditions can affect the microbial fitness and community composition. For instance, Baleeiro et al. showed that small air contamination can be detrimental to lactic acid -based chain elongation. In experiments that were conducted in bioreactors, which were inoculated with open cultures, the authors showed a strong inhibition of caproic acid production and methanogenesis upon continuous exposure to low oxygen concentrations, although no clear effect was observed on butyric acid production. The authors pointed out a possible interaction between O_2_ and H_2_ (which was recirculated), leading to the potential formation of reactive oxygen species (ROS). Upon recovering complete anaerobiosis, caproic acid and methane production recovered to values close to those of a control experiment. This indicated a reversible inhibition, even after >25 days of exposure. A last interesting observation of this study was that H_2_ availability also led to iso-butyric acid production, a possibility that we already hinted on earlier (Petrognani et al., 2020). The authors also proposed micro-aerophilic conditions as a possible strategy to steer lactate conversion to the production of propionic acid, since propionic acid producers are hypothesized to be aero-tolerant.

The impact of different pH levels on community composition and product inhibition was also investigated in this chain elongation special topic https://doi.org/10.3389/fbioe.2021.693030. The enrichment of ethanol-based chain elongating bacteria for two pH levels yielded communities that were dominated by *Clostridium kluyveri*, which is in agreement with what has been reported in other (meta)studies (Candry and Ganigué, 2021). Despite showing similar elongation stoichiometries, lower conversion to caproic acid (71 ± 6 vs. 30 ± 5% on electron basis) were reported at pH 7 compared 5.5, respectively, which differs with the observations of Candry and co-workers (Candry et al., 2020a). Interestingly, specific mildly acidic pHs led to a decrease in the biomass yield due to weak acid uncoupling inhibition, but a significant increase in the biomass-specific substrate uptake rate, which authors attribute to organisms employing catabolic overcapacity to deal with energy losses associated to product inhibition. Ultimately, this study also shows the potential of sequencing batch reactors as a tools for bioprocess characterization, since the dynamics revealed in batch cultivation can provide deeper insights than continuous-stirred tank reactors.

#### 1.2.3 Feedstock Utilization and Platforms Development

Agler et al. (2012b) performed a study with pretreated lignocellulosic fiber to produce mainly butyric acid at thermophilic conditions from hydrolysates, however, some caproic acid was also produced (this study was performed before Agler et al.‘s other studies to primarily produce caproate). The study by Ingle et al. in this Research Topic used pretreated lignocellulosic fiber from cow manure at mesophilic conditions. Similar fermentation outcomes were reported compared to*.* (Agler et al., 2012b), including a predominant butyric acid product as the carboxylic acid, a superior dilute-acid pretreatment method, and pertinent lactic acid producing and consuming populations. The intermediate for chain elongation was likely extracellular lactic acid, but a caveat must be placed here because solely 16S rRNA gene sequencing surveys are not sufficient to declare function. This is especially true for hydrolysates that contain xylose and glucose. The microbiome-member *Caproiciproducens* spp., for example, can utilize sugars for caproate or lactic acid production (Esquivel-Elizondo et al., 2021). However, the network and redundancy analysis in Agler et al. (2012b) and the redundancy analysis in Ingle et al. make it more likely that lactic acid is the pertinent electron donor for chain elongation from hydrolysates, especially towards butyric acid production. Thus, it seems less likely that sugars were a direct electron donor for chain elongation.

Several studies from two different research groups had performed bioreactor experiments to convert thin stillage into caproic acid and other fermentation products. Thin stillage is the supernatant of ethanol beer from dry mills after ethanol removal by distillation, and thus ethanol would not be the obvious electron donor for chain elongation. Constituents of thin stillage are glycerol, carbohydrates (including C5 and C6 sugars), lactic acid, and protein as used by Fortney et al. in this Research Topic. Andersen et al. (2015) had combined bioreactors with electrochemical product extraction to observe caproic acid production and they had proposed lactic acid as a possible electron donor for such chain elongation to caproic acid. The small concentrations of ethanol were often not even removed by the microbiome. Next, a similar group of authors studied the effect of product toxicity on the microbial ecology with thin stillage as the substrate (Andersen et al., 2017) to find advantages in product toxicity to enrich certain community members. A different research group, Scarborough et al. (2018b) also found caproic acid production from thin stillage and identified lactic acid as a likely electron donor for such chain elongation, while excluding ethanol as electron donor in another study by metagenomic analysis (Scarborough et al., 2018a). Importantly, Scarborough et al. (2020) in a metabolic modeling effort raised concerns about lactic acid as the electron donor for caproic acid production and suggested that sugars would be the main electron donor, while lactic acid would be the electron donor for chain elongation to butyric acid. This knowledge base was taken into consideration in the thin stillage study by Fortney et al. in this Research Topic. The operating conditions were altered to change the fermentation product spectrum from SCCAs and MCCAs to either succinic acid (a periodic short SRT) or lactic acid and propionic acid (thermophilic conditions). The authors also discussed that the largest pool of carbon in substrate came from glycerol, and discussed the microbiome members.

#### 1.2.4 Product Separation

The first two studies with a clear goal to develop open-culture caproic acid production as an industrial chain elongation biotechnological production platform in the 2010s had utilized two different product extraction philosophies. First, Steinbusch et al. (2011) had operated the bioreactor system at a neutral pH without in-line product extraction. Thus, product extraction would need to occur as a post-treatment step with the fermentation broth. The potential strategy for this by ChainCraft in Amsterdam, the Netherlands is presented in an evaluation paper by the European Food Safety Authority (Ricci et al., 2018). Second, Agler et al. (2012a) utilized in-line product extraction to recover caproic acid from the mixed liquor at a mildly acidic pH. An existing membrane-based liquid-liquid extraction (*i.e.*, pertraction) technology was adapted to prevent emulsification with the complex substrate that consisted of corn-to-ethanol fermentation beer (Schlosser et al., 2005). The same strategy is continued by Capro-X in Ithaca, NY (Capro-X, 2022). The study by Braune et al. in this Research Topic is one of the first published post-treatment systems for caproic acdi and caprylic acid concentration into a solvent. A further distillation step would be needed to separate the product from the solvent (Saboe et al., 2018). The authors chose a counter-current liquid-liquid extraction tower system without membranes. To prevent emulsification, they included: **1)** a filter press to remove solids from the fermentation broth; and **2)** an ultrafiltration membrane system to remove suspended particles, microbes, macromolecules, and colloids from the solids-free fermentation broth. The filter press and ultrafiltration steps resulted in caproic acid and caprylic acid losses of 21% and 13%, respectively, due to losses of fermentation broth with soluble caproic and caprylic acid. The extraction efficiencies for the liquid-liquid extraction were 85% and 97% for caproic acid and caprylic acid, respectively, and very similar to the in-line extraction efficiencies of pertraction (Agler et al., 2012b). The authors suggested utilizing different solids-removal technologies with lower losses of fermentation broth.

In-line product extraction of caproic acid and caprylic acid had been performed ever since the early 2010s (Agler et al., 2012a). Until recently, the forward-membrane modules were placed outside the bioreactor. Similar to membrane bioreactors, however, they can also be placed inside bioreactors. The study by Xu et al. in this Research Topic did exactly that: it successfully moved the forward membranes inside the bioreactor and then ascertained whether special anti-fouling measures were necessary. The hydraulic pressure within the bioreactor due to the column height of mixed liquor was used to prevent the solvent from leaking out of the hollow-fiber membrane. Several different measures to prevent biofouling of the membranes were tested in this study, including an increased hydraulic upflow velocity via liquid recycling and an increased biogas recycling rate. The former was found to be more efficient, but extra care needs to be taken in the future. A considerably higher upflow velocity changed the chain-elongating microbiome into a methanogenic microbiome, transitioning the system into an anaerobic digester. Indeed, a 16S rRNA gene sequencing approach identified an increase in the density of the genera *Methanobacterium* and *Methanobrevibacter* in the biomass after the change in the upflow velocity. Apparently, H_2_ was lost during the recirculation of mixed liquor due to diffusion, changing the limiting factor of methanogenesis from CO_2_ to H_2_. A resulting loss of the H_2_ partial pressure in the system removed the thermodynamic constraint of oxidizing ethanol into acetic acid under anaerobic conditions, eventually leading to a large increase in methane production due to syntrophic acetic acid oxidation in the presence of hydrogenotrophic methanogens.

### 1.3 Future Perspectives

Chain Elongation is an emerging research field, which is rapidly explored by industries for their commercial application possibilities (Strik et al., 2020). There is a need to understand the potential impact of chain elongation whereby societal as well as environmental aspects, and a life cycle analysis, are to be included. Many research directions and key challenges are already provided in several review papers (Angenent et al., 2016; Cavalcante et al., 2017; Wu et al., 2019a; Baleeiro et al., 2019; De Groof et al., 2019). We are excited to see how the research field is exploring in the future. To find out, we are organizing the second ICEC meeting this fall and a subsequent new special issue in frontiers.
